# Social phenomena following the tobacco tax increase in South Korea: Lessons and policy implications

**DOI:** 10.18332/tid/84891

**Published:** 2018-05-30

**Authors:** Myung-Bae Park, Eun W. Nam, Hye L. Lee, Kyoung Soo Hong, Yumi Oh

**Affiliations:** 1 Department of Gerontal Health and Welfare, Pai Chai University, Daejeon, Republic of Korea; 2 Department of Health Administration, Yonsei University, Wonju, Republic of Korea; 3 Korea Human Resource Development Institute for Health and Welfare, Osong Health Technology Administration Complex, Cheongju-si, Republic of Korea; 4 Korea Health Promotion Institute, Seoul, Republic of Korea

**Keywords:** tobacco control, tobacco tax, health policy, National Health Promotion Fund, South Korea

## Abstract

**INTRODUCTION:**

This paper reviews the trial and error occurring before an increase in cigarette prices and the subsequent effects of this in South Korea. In addition, we introduce the social phenomena that occur as a result of an increase in tobacco tax, and propose effective strategies and principles that need to be taken into account before increasing cigarette prices.

**METHODS:**

We compared changes to smoking rates before and after the increase in cigarette prices. To investigate the changes that occurred before South Korea’s increase in tobacco tax, we first analysed the state of cigarette consumption and then the change in smoking rates.

**RESULTS:**

The increase in cigarette prices caused an immediate backlash from smokers, particularly low-income groups and those claiming tax inequality. In particular, the sales of electronic nicotine delivery systems (ENDS) increased dramatically and the lower price marketing of tobacco companies led to short-term market share increases. As expected, smoking rates in South Korea decreased. However, because the price increase was not sufficient to encourage widespread smoking cessation, the decrease in smoking rates was not significant.

**CONCLUSIONS:**

Because the primary objective of the cigarette pricing policy was not designed to promote public health, by reducing smoking rates, it received public criticism. To avoid public criticism, the government must emphasize and convince the public that the primary objective of increasing cigarette prices is to protect public health through a decline in smoking rates. Ideally, health authorities should play a leading role in formulating tobacco tax policy.

## INTRODUCTION

Increasing the price of cigarettes is one of the most effective ways to reduce smoking rates^1^. In January 2015, an important year that marked the tenth anniversary of the Framework Convention on Tobacco Control (FCTC) devised by the World Health Organization (WHO), South Korea enforced a nearly two-fold increase of KRW 2000 on the price of cigarettes. This was its first increase after the price increase of KRW 500 in 2004.

South Korea’s smoking prevalence and cigarette consumption per capita is ranked high in the world^2,3^, and a need for strong anti-smoking policies has continually been raised as a national public health issue. Before the recent increase in cigarette prices, the South Korean government had implemented numerous anti-smoking policies aiming to reduce the high smoking rate. Since 2005, public health centers nationwide have been operating smoking cessation clinics. In 2012, some public facilities that previously included specific smoking areas needed to designate their entire buildings as non-smoking. Moreover, in 2013, the number of advertisements permitted for each type of cigarette product was decreased from 60 to 10 per year. In 2014, the use of misleading phrasing in advertising was restricted, and electronic and smoke-free cigarettes were required to display warnings^4^.

Until December 2014, the retail price per 20 cigarettes in South Korea was KRW 2500 (USD 2.2), which was less than half of the OECD average (USD 6), and was lower than the averages of 19 Asia-Pacific countries^5^. Because smoking is one of the most important health-risk factors, reducing smoking rates is an important way to improve national and global public health. An increase in cigarette prices is generally considered the most effective anti-smoking strategy^6,7^, however, for numerous reasons, many countries find it difficult to implement tobacco tax increases. The purpose of this study is to propose effective strategies and principles to overcome the barriers to increasing cigarette prices. Based on this assessment and the fundamental strategies of other countries planning to implement cigarette pricing policies, we propose suggestions to the South Korean government regarding cigarette pricing.

## METHODS

To investigate the social and political changes taking place before an increase in tobacco tax, we conducted qualitative analysis, additionally, we used quantitative data to explore social phenomena following a change in the tobacco tax policy.

## RESULTS

Smoking cessation due to increased cigarette prices begins when the price exceeds a smoker’s WTP (willingness to pay). However, smoking cessation in response to increased prices is an extreme option for smokers to choose because there are many ways to continue smoking, such as switching to a cheaper option or reducing other expenses^8^. Currently, legal tobacco products in South Korea include cigarettes, pipes, cigars, chewing gum, snuff, water pipes, rolled cigarettes, heat-not-burn, and electronic nicotine-delivery systems (ENDS). Sales of ENDS rose dramatically because, in the face of the increased price of cigarettes, companies that produced ENDS actively marketed their products by describing them as less harmful to health or relatively more economical.

Individual pre-rolled cigarettes and loose tobacco, which the user rolls directly into cigarette papers, also are available to reduce consumers’ financial burden. Because it is illegal to sell individual cigarettes in South Korea, this phenomenon is becoming a social issue. Meanwhile, the increased price of cigarettes has driven up the cost of most cigarette brands to about KRW 4500. British American Tobacco and Japan Tobacco International engaged in tactical marketing to increase their market share, which reduced profits and supplied some products at lower prices between about KRW 3500 and KRW 4000. They also sold packs of 14 cigarettes for KRW 3000. This strategy led to a short-term increase in the market share of these two companies during the first half of 2015^9^.

In january 2015, the retail price of a pack of cigarettes was increased by KRW 2000 (from KRW 2500 to KRW 4500). Consequently, this resulted in total tax increase by KRW 1758. Within that tax, a special consumption tax (SCT) had the highest increase at KRW 594, followed by the NHPF (National Health Promotion Fund) tax increase of KRW 488, the tobacco consumption tax increase of KRW 366, and the value-added tax increase of KRW 182 (Table 1).

**Table 1:** Structure of cigarette pricing. (Unit: KRW, 20 cigarettes per pack)

	*Before (%) (~Dec. 2014) (A)*	*After (%) (Jan. 2015~) (B)*	*Price and portion gap (C)=(B)-(A)*	*Property*
**Retail price**	2500 (100.0)	4500 (100.0)	2000 (-)	
**Production cost** (including profit and margin)	950 (38.0)	1182 (26.3)	232 (-11.7)	Tobacco company
Total tax	1550 (62.0)	3318 (73.7)	1758 (11.7)	
Tobacco consumption tax	641 (25.6)	1007 (22.4)	366 (-3.3)	Local government
Local education tax	321 (12.8)	443 (9.8)	122 (-3.0)	Local government
National Health Promotion Fund	354 (14.2)	841 (18.7)	488 (4.5)	Ministry of Health and Welfare
Charges on waste tax	7 (0.3)	24 (0.5)	6 (0.2)	Ministry of Environment
Value added tax	227 (9.1)	409 (9.1)	182 (0.0)	Ministry of Strategy and Finance
Special consumption tax	-	594 (13.2)	594 (13.2)	Ministry of Strategy and Finance

Source: Ministry of Strategy and Finance (2015). Note that the table was reconstructed. USD 1 = KRW 1135; EUR 1 = KRW 1216 (21 April 2017)

South Korea ultimately succeeded in increasing the price of cigarettes, which is the most effective anti-smoking strategy^6,7^. Following the increase, taxes accounted for 73.7% of the retail price, which is close to the minimum threshold of 70% recommended by the WHO. It is also higher than the global average of 59.4%, and it is the highest percentage among the Western Pacific countries^10^.

Cigarette prices in South Korea (USD 3.79 per pack) are higher than the global mean (USD 3.58), but lower than the mean price in high-income (USD 5.53)^11^ and OECD (USD 6) countries^5^. Therefore, South Korean smokers might have expected to be affected by the price increase. According to a Government report, smoking rates in South Korea have decreased. The results of the study found that the smoking rates of men and women were 39.3% and 5.5%, respectively, lower than the official rates reported for 2015^12^. However, because the amount of increase was not sufficient to encourage widespread smoking cessation, the decrease in smoking rates was not large.

## DISCUSSION

Although the price increase is an example of implementation of the anti-smoking policy for socially vulnerable individuals, such as youths, the elderly, and low-income groups^6,13-15^, it has instead sparked debate about the supply of low-cost cigarettes to elderly and poor people.

The South Korean government’s strategy for increasing revenue by raising the price of cigarettes might seem obvious considering similar approaches in many other countries^16^. However, criticism that the government’s policy was designed to increase tax revenue has been quite strong and has led to the near failure of the tobacco tax policy. This was caused by the government’s inability to convince the public that the fundamental goal of the price increase was to promote public health. The following suggestions are intended to help other countries avoid making this mistake when implementing similar policies.

### Policy implications

First, the government must emphasize that its primary reason for increasing the price of cigarettes is to promote public health through lower smoking rates. When this altruistic objective is unconvincing, it becomes difficult to persuade the public that increased prices will provide this benefit. Thus, health authorities should play a central role in the implementation of price increase policies. In addition, health authorities need to gain support from health-related citizens’ organizations, such as anti-smoking movements and the medical association, as this will have a positive impact.

Second, measures are needed that minimize the potential adverse effects of a price increase, which could include illegal product circulation and increased efforts by the tobacco industry to promote substitutes, such as e-cigarettes or down-market cigarettes.

Third, the government must continue to fund anti-smoking projects. If the government were to increase its funding of anti-smoking projects under false pretenses, as was done in 2004, and then gradually decrease its funding, the continuity of anti-smoking policies would be interrupted, which would create a considerable barrier to the implementation of future tobacco tax policies. Other countries currently considering a price increase would benefit from this observation.

## CONCLUSIONS

Governments must emphasize and convince the public that the primary objective of increasing cigarette prices is to protect public health through a decline in smoking rates. Ideally, health authorities in a country should play a leading role in the formulation of tobacco tax policy.
